# Case report: A case of ITP-like thrombocytopenia induced by denosumab

**DOI:** 10.3389/fphar.2026.1723393

**Published:** 2026-04-29

**Authors:** Shijing Chen, Xiahong Lin

**Affiliations:** Department of Geriatrics, The Seventh Affiliated Hospital, Sun Yat-sen University, Shenzhen, Guangdong, China

**Keywords:** denosumab, immune thrombocytopenia, adverse drug reaction, osteoporosis, case report

## Abstract

Denosumab has been widely utilized in the management of osteoporosis; however, thrombocytopenia as an adverse reaction associated with denosumab has been infrequently reported, and its underlying mechanism is not yet fully understood. We present a retrospective analysis of a rare case of suspected immune-mediated thrombocytopenia resembling immune thrombocytopenia (ITP) in a patient with primary osteoporosis following denosumab treatment, along with a comprehensive review of the relevant literature to investigate the potential mechanisms of denosumab-induced ITP-like thrombocytopenia. This case underscores the need for routine platelet monitoring during clinical use of denosumab and serves as a pharmacovigilance signal, providing a basis for future drug safety assessments.

## 1 Introduction

Osteoporosis is a systemic skeletal disorder characterized by reduced bone mass and deterioration of bone microarchitecture, leading to a significantly increased risk of fractures. It represents one of the primary causes of morbidity and mortality among older adults and has emerged as a critical global public health concern (Qaseem et al., 2023). Denosumab competitively inhibits the interaction between Receptor Activator of Nuclear Factor-kappa B (RANK) and its ligand (RANKL), thereby suppressing osteoclastic bone resorption and reducing the risk of skeletal fractures (Boyle et al., 2003; Delmas, 2008). Compared with traditional bisphosphonates, denosumab offers advantages including a rapid onset of action, convenient subcutaneous administration, and a favorable overall safety profile. It is widely used in the management of postmenopausal osteoporosis, male osteoporosis, glucocorticoid-induced osteoporosis, and osteoporosis related to cancer (Cummings et al., 2009; Langdahl et al., 2015; Nakamura et al., 2014; Saag et al., 2018; Yanbeiy and Hansen, 2019; Ellis et al., 2008). The safety profile of denosumab is generally favorable, with hematological adverse effects—such as anemia, neutropenia, and thrombocytopenia—reported only in certain cancer patient populations (Raje et al., 2018; Fizazi et al., 2011; Henry et al., 2011; Stopeck et al., 2010). Immune thrombocytopenia (ITP) is a hemorrhagic disorder characterized by autoantibody-mediated platelet destruction and impaired platelet production (Neunert et al., 2019). The condition typically presents with mucocutaneous bleeding and thrombocytopenia. In recent years, with the increasing clinical application of biological agents, there has been a gradual rise in cases of ITP associated with monoclonal antibodies. We report a case of a patient with primary osteoporosis who developed ITP-like thrombocytopenia following denosumab therapy, aiming to emphasize the importance of monitoring the hematologic adverse effects of denosumab.

## 2 Case presentation

The patient was a 73-year-old female diagnosed with primary osteoporosis complicated by an old pathological fracture of the thoracic spine over 1 year ago. As she was asymptomatic, no specific treatment was initiated at that time. Subsequently, denosumab (Prolia, 60 mg subcutaneously every 6 months) was started for osteoporosis management. The second dose was administered 7 months ago. Four months ago, the patient developed scattered spontaneous petechiae and ecchymoses on the abdomen and extremities, along with bilateral conjunctival hemorrhage. Although the conjunctival hemorrhage resolved after more than 10 days, the cutaneous petechiae and ecchymoses persisted. The patient did not seek medical attention or undergo hematologic evaluation at that time. One month ago, she received the third scheduled dose of denosumab, following which she developed an increased number and extent of skin ecchymoses, accompanied by fatigue. She then presented to our hospital for further evaluation. The patient denied any special dietary history, family history, or history of genetic disorders. On admission, physical examination revealed scattered punctate and patchy purpuric petechiae and ecchymoses on the abdomen and extremities (as shown in Supplementary Figure S1). The patient’s demographic characteristics, comorbidities, concomitant medications, and other laboratory data are summarized in Table 1.

**TABLE 1:** Clinical characteristics and laboratory data of the patient.

Category	Parameter	Finding	Timepoint
Demographics	Age/Sex	73 years/Female	At presentation
Comorbidities	Primary osteoporosis	With pathological fracture of thoracic spine	Diagnosed >1 year prior
​	Type 2 diabetes mellitus	With atherosclerosis, peripheral neuropathy	Chronic
​	Coronary atherosclerotic heart disease	Status post-PCI	Chronic
​	Hypertension, hyperlipidemia, atrial premature beats, fatty liver	Present	Chronic
Concomitant medications	Aspirin, insulin glargine, semaglutide, metformin, acarbose, chiglitazar, losartan, rosuvastatin	Regular doses	Long-term use
Other hematologic parameters	Platelet count	10 × 10^9^/L	At admission
​	Hemoglobin	149 g/L	At admission
​	White blood cell count	9.87 × 10^9^/L	At admission
Coagulation	PT, APTT	Within normal limits	At admission
Liver and kidney function	ALT, AST, creatinine, BUN	Within normal limits	At admission
Electrolytes	Sodium, potassium, calcium, phosphorus	Within normal limits	At admission
Exclusion of other causes			
- Infectious workup	HIV, HBV, EBV, CMV	Negative	During admission
- Autoimmune workup	ANA, anti-dsDNA, ANCA, RF, anti-cardiolipin antibodies	Negative	During admission
- Nutritional	Vitamin B12, folate	Normal	During admission
- Thyroid function	TSH, free T3, free T4	Normal	During admission
- Tumor markers	CEA, CA19-9, etc.	Negative	During admission
- Lymphoproliferative disorders	Serum protein electrophoresis, serum and urine immunofixation	Normal	During admission

ALT, alanine aminotransferase; AST, aspartate aminotransferase; aPTT, activated partial thromboplastin time; BUN, blood urea nitrogen; CMV, cytomegalovirus; EBV, Epstein–Barr virus; HBV, hepatitis B virus; HCV, hepatitis C virus; HIV, human immunodeficiency virus; PCI, percutaneous coronary intervention; PT, prothrombin time; TSH, thyroid-stimulating hormone.

At admission (December 2024), the patient’s platelet count was 10 × 10^9^/L, whereas it had been normal over 1 year prior (November 2023). Red blood cell and white blood cell counts, as well as liver and kidney function tests and coagulation parameters, showed no significant abnormalities. Peripheral blood smear analysis revealed a marked reduction in platelet count with no significant morphological abnormalities. Subsequent bone marrow aspiration and biopsy demonstrated active hematopoietic proliferation, increased megakaryocytes with impaired maturation, and a markedly decreased platelet count despite normal platelet morphology, findings suggestive of ITP (Author Anonymous, 2025). However, testing for platelet-specific autoantibodies was negative, which did not fully confirm the diagnosis of ITP. We then systematically excluded other common causes of thrombocytopenia, including rheumatologic diseases, lymphoproliferative disorders, HIV, hepatitis B, EBV, CMV, thyroid diseases, malignancies, as well as common culprit drugs such as antibiotics, anticoagulants, antineoplastic agents, and immunomodulatory agents.

**FIGURE 1:**
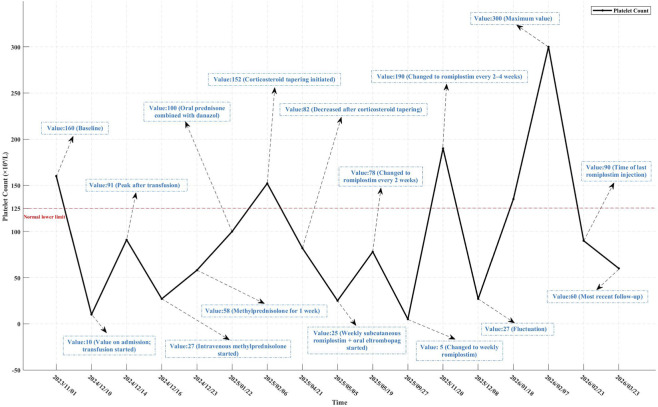
Platelet count over time.

Treatment began with an emergency transfusion of three units of platelets. The platelet count transiently rose to a peak of 91 × 10^9^/L but declined to 27 × 10^9^/L 2 days later, a pattern consistent with peripheral destruction. Intravenous methylprednisolone (60 mg once daily) was promptly initiated, and the platelet count gradually increased to 58 × 10^9^/L after 1 week. The patient was then switched to oral prednisone combined with danazol, and the platelet count reached a peak of 152 × 10^9^/L in February 2025. During glucocorticoid tapering, the platelet count fell to 82 × 10^9^/L in April 2025 and further dropped to 25 × 10^9^/L in early May 2025, prompting second-line therapy with weekly subcutaneous romiplostim (0.25 mg) combined with oral eltrombopag. The platelet count initially rose to 78 × 10^9^/L, after which the romiplostim dosing interval was extended to every 2 weeks. However, platelet counts became unstable, with a nadir of 5 × 10^9^/L—likely attributable to poor medication adherence. Upon resumption of weekly romiplostim plus eltrombopag, the platelet count increased to 190 × 10^9^/L. Subsequently, the romiplostim interval was gradually extended to every 2–4 weeks. Although a transient decrease to 27 × 10^9^/L occurred during this period, platelet counts subsequently improved, reaching a maximum of 300 × 10^9^/L in early February 2026. At the most recent follow-up (March 2026), due to the prolonged romiplostim dosing interval, the platelet count had decreased to 60 × 10^9^/L, yet remained within a relatively safe range. The platelet course is summarized in Figure 1.

Regarding the subsequent management of osteoporosis, given the close association between the patient’s ITP and denosumab therapy, we recommended discontinuing denosumab. However, in light of the patient’s history of osteoporosis with pathological fracture, abrupt discontinuation posed risks. Therefore, 6 months after denosumab cessation and with a stable platelet count, we suggested annual intravenous infusions of 5 mg zoledronic acid for osteoporosis management. Over a subsequent 1-year follow-up, no new fractures occurred, and bone mineral density remained stable.

## 3 Discussion

Primary ITP is an acquired autoimmune bleeding disorder primarily characterized by isolated thrombocytopenia without a clear cause. Its main pathogenesis involves the loss of immune tolerance to platelet autoantigens, leading to accelerated platelet destruction and impaired megakaryocyte maturation (Neunert et al., 2019; Zufferey et al., 2017). Given the relatively low sensitivity of platelet-specific antibodies, the diagnosis of ITP is typically established by excluding bone marrow disorders and other common causes of thrombocytopenia. This patient presented with isolated thrombocytopenia and cutaneous and mucosal bleeding. Bone marrow examination revealed megakaryocytosis with impaired maturation; these features overlap with those of primary ITP. Considering the patient’s response to treatment—platelets were rapidly depleted following transfusion, and there were no signs of hemolysis or splenomegaly—this suggests peripheral destruction. Furthermore, drug therapy for ITP was effective, a presentation consistent with primary ITP.

However, the following findings do not support a diagnosis of primary ITP: (a) Negative results for platelet-specific autoantibodies; although a negative result does not completely rule out ITP, it reduces the likelihood of classic autoimmune-mediated platelet destruction. (b) Impaired megakaryocyte maturation in the bone marrow: In typical ITP or classic drug-induced immune thrombocytopenia (DITP), bone marrow megakaryocytes are typically compensatorily increased; although maturation defects may also occur, the possibility of bone marrow toxicity cannot be overlooked. (c) The temporal association between clinical symptoms and the administration of denosumab, coupled with the fact that the patient experienced a rechallenge phenomenon after inadvertently receiving a third injection of denosumab, strongly suggests that this drug is a triggering factor. We used the Naranjo Adverse Drug Reaction Probability Scale (Naranjo et al., 1981) for assessment (as shown in Supplementary Table S1), and the patient scored 6, corresponding to a “probable” association. However, caution is warranted when applying the Naranjo scale to biologics such as denosumab. The scale relies heavily on dechallenge and rechallenge, and drug rechallenge is often ethically unfeasible in clinical practice. Moreover, the scale does not incorporate immunological confirmation, such as detection of drug-dependent antibodies (DDAbs). To supplement the assessment, we referred to the WHO-UMC causality classification system (NTEP, 2017) (as shown in Supplementary Table S2) and, based on a reasonable temporal relationship, exclusion of alternative etiologies, and response to immunosuppressive therapy, rated the causality as “probable/likely”. These findings indicate that the patient most likely DITP induced by denosumab.

DITP is a clinical syndrome characterized by isolated thrombocytopenia resulting from drug exposure that triggers an immune response, leading to accelerated platelet destruction or impaired platelet production (Bakchoul and Marini, 2018). Based on the underlying pathogenesis, DITP can be classified into two main categories: non-immune-mediated and immune-mediated. In some DITP patients, drug-dependent antiplatelet antibodies bind non-covalently to specific platelet antigens via their Fab regions, accelerating platelet destruction (Aster and Bougie, 2007; Arnold et al., 2013). In other cases, drugs may bind to the platelet surface and induce conformational changes in surface proteins, exposing new epitopes that stimulate the formation of antiplatelet antibodies (Burgess et al., 1998). Unfortunately, due to limited clinical experience and technical resources, we were unable to perform DDAbs testing. It should be noted that, given the diverse pathogenesis of DITP, the causative agent may be a drug metabolite rather than the drug itself; consequently, DDAbs are generally difficult to detect in the laboratory (Bougie and Aster, 2001; Bougie et al., 2007). Even a negative test result cannot completely rule out the possibility of DITP (Leach et al., 1997).

Compared with previously reported cases, there have been very few reports of denosumab-associated thrombocytopenia. Denosumab is primarily available in two formulations: Prolia (60 mg) and XGEVA (120 mg). Existing data primarily come from oncology trials in which patients received XGEVA (120 mg every 4 weeks). In a Phase III trial of patients with multiple myeloma, 24% of those treated with XGEVA developed thrombocytopenia (Raje et al., 2018). However, these cases were typically attributed to disease-related bone marrow suppression or concomitant chemotherapy. To our knowledge, there have been no reported cases of ITP suspected to be induced by denosumab in patients treated with Prolia (60 mg every 6 months) for osteoporosis.

A small number of case reports have described immune-mediated thrombocytopenia associated with other monoclonal antibodies, such as the immune checkpoint inhibitors nivolumab and ipilimumab, and the TNF-α inhibitors infliximab and adalimumab (Marini et al., 2022; Casanova et al., 2012). Currently, it is believed that DITP induced by these biologics promotes platelet destruction through the reactivation of exhausted CD4^+^ and CD8^+^ T cells, leading to the breakdown of thymic immune tolerance and facilitating autoimmune platelet destruction, or by inducing the production of anti-platelet antibodies; however, the exact pathophysiological mechanism remains unclear (Moore et al., 2024). In these cases, thrombocytopenia typically resolves after discontinuation of the medication. What distinguishes this case is that, despite discontinuation of denosumab, the patient’s thrombocytopenia persisted for over a year, suggesting that immune homeostasis may have been disrupted more persistently. However, some studies have suggested that in certain cases of biologic-induced DITP, autoantibodies can bind to platelets even in the absence of the drug, leading to a continued decline in platelet counts even after discontinuation of the relevant medication, particularly for drugs with long half-lives.

Based on the clinical features of this case—including a clear temporal association with drug exposure, a rapid decline in platelet count following a blood transfusion, a response to corticosteroid therapy (suggesting peripheral immune destruction), impaired megakaryocyte maturation in the bone marrow, and a treatment-dependent response to the second-line drug TPO-RA (suggesting impaired platelet production)—we believe that the pathophysiological mechanism is not limited to classic DITP alone, but rather involves the coexistence of peripheral immune destruction and impaired platelet production. Therefore, we ultimately diagnosed this case as denosumab-induced ITP-like thrombocytopenia.

Denosumab is a fully humanized monoclonal antibody that targets RANKL, a key regulator of osteoclastogenesis. In addition to its effects on bone metabolism, the RANKL/RANK signaling pathway plays a significant role in the immune system, including the survival of dendritic cells, the activation of macrophages and T cells, and the induction of tolerance (Kong et al., 1999). RANKL inhibition by denosumab may theoretically disrupt thymic tolerance, leading to the sustained activation of autoreactive T cells, which could explain the clinical feature of prolonged thrombocytopenia lasting over 1 year after discontinuation of the drug in this case. Recent studies have shown that megakaryocytes can express RANKL, and the NF-κB signaling pathway plays a key regulatory role in megakaryocyte differentiation and platelet production. By inhibiting RANKL, denosumab may have affected the interaction between immune cells and the NF-κB signaling pathway, thereby affecting megakaryocyte maturation and function (Kikuchi et al., 2023; ZHANG and YAN, 2020). This appears to account for the impaired megakaryocyte maturation observed in the bone marrow biopsy of this case, as well as the treatment-dependent response of platelets to TPO-RA.

This case has the following practical implications: (a) It underscores the necessity of routine complete blood count monitoring for patients receiving denosumab (especially those on long-term therapy). (b) When patients receiving denosumab develop thrombocytopenia, particularly in the absence of other identifiable causes, discontinuation of the drug should be considered. However, as demonstrated in this case, platelet counts may not return to normal immediately after discontinuation; sustained monitoring is required, and immunosuppressive therapy may be necessary in some cases. (c) Re-exposure to denosumab is not recommended for patients who have experienced a reaction that is likely to be immune-mediated. (d) Switching to zoledronic acid therapy is feasible. In this case, after a 6-month washout period and confirmation of stable platelet counts, the patient was switched from denosumab to zoledronic acid. During the 1-year follow-up period, thrombocytopenia did not recur, and no new fractures occurred. In conclusion, this case serves as a pharmacovigilance signal rather than definitive mechanistic proof, highlighting the necessity for enhanced monitoring and systematic reporting of hematologic adverse events associated with biologics. Future studies with larger sample sizes are needed, ideally incorporating immunological characterization (e.g., T-cell assays, drug-specific antibody testing), to elucidate the underlying mechanisms.

## 4 Conclusion

In this case report, we describe that denosumab induced persistent immune-mediated thrombocytopenia resembling primary ITP, which persisted even after drug discontinuation, suggesting possible concomitant myelosuppression. However, due to the inherent limitations of a single case report, larger-sample studies are needed to validate this causal relationship. This case highlights the necessity of routine platelet monitoring during clinical use of denosumab and serves as a pharmacovigilance signal rather than definitive mechanistic proof. Further studies are required to elucidate the underlying mechanisms.

### 4.1 Patient perspective

Due to the patient’s advanced age and prolonged, unstable illness, the family spoke on her behalf. They described immense psychological stress and anxiety from over a year of thrombocytopenia, particularly when platelet counts repeatedly dropped after each treatment wore off. They expressed gratitude for the team’s clear communication and follow-up. The family consented to publication to raise awareness of rare adverse drug reactions and long-term recovery challenges.

## Data Availability

The original contributions presented in the study are included in the article/Supplementary Material, further inquiries can be directed to the corresponding author.

## Supplementary material

SUPPLEMENTARY FIGURE S1 Cutaneous petechiae and ecchymoses: **(A)** Hand; **(B)** Foot; **(C)** Abdomen.
