# Supplementary material for: Case report: A case of ITP-like thrombocytopenia induced by denosumab
Source: Front Pharmacol. 2026 Apr 29;17:1723393. doi: 10.3389/fphar.2026.1723393 (PMC13167528; doi:10.3389/fphar.2026.1723393)
Supplement: Supplementary file 1 [file Table1.docx]

| **Question** | **Yes** | **No** | **Not known or not done** | **Score** |
| --- | --- | --- | --- | --- |
| **1. Are there previous conclusive reports on this reaction?** | **+1** | **0** | **0** | **0** |
| **2. Did adverse event appear after the suspected drug was given?** | **+2** | **-1** | **0** | **+2** |
| **3. Did the adverse reaction improve when the drug was discontinued or a specific antagonist was given?** | **+1** | **0** | **0** | **0** |
| **4. Did the adverse reaction appear when the drug was readministered?** | **+2** | **-1** | **0** | **+2** |
| **5. Are there alternative causes that could have caused the reaction?** | **-1** | **+2** | **0** | **+2** |
| **6. Did the reaction reappear when a placebo was given?** | **-1** | **+1** | **0** | **0** |
| **7. Was the drug detected in any body fluid in toxic concentrations?** | **+1** | **0** | **0** | **0** |
| **8. Was the reaction more severe when the dose was increased, or less severe when the dose was decreased?** | **+1** | **0** | **0** | **0** |
| **9. Did the patient have a similar reaction to the same or similar drugs in any previous exposure?** | **+1** | **0** | **0** | **0** |
| **10. Was the adverse event confirmed by any objective evidence?** | **+1** | **0** | **0** | **0** |
| **Score** | **6，Probable ADR** | | | |

Note:

Doubtful ADR (≤0): The reaction was likely related to factors other than a drug.

Possible ADR (1 to 4): The reaction followed a temporal sequence after a drug, possibly followed a recognized pattern to the suspected drug, and could be explained by characteristics of the patient’s disease.

Probable ADR (5 to 8): The reaction followed a reasonable temporal sequence after a drug, followed a recognized response to the suspected drug, was confirmed by withdrawal but not by exposure to the drug, and could not be reasonably explained by the known characteristics of the patient’s clinical state.

Definite ADR (≥9): The reaction (1) followed a reasonable temporal sequence after a drug, or a toxic drug level had been established in body fluids or tissues; (2) followed a recognized response to the suspected drug; and (3) was confirmed by improvement on drug withdrawal and reappearance upon re‑exposure.
