# Supplementary figures and images for: Case report: A case of ITP-like thrombocytopenia induced by denosumab
Source: Front Pharmacol. 2026 Apr 29;17:1723393. doi: 10.3389/fphar.2026.1723393 (PMC13167528; doi:10.3389/fphar.2026.1723393)

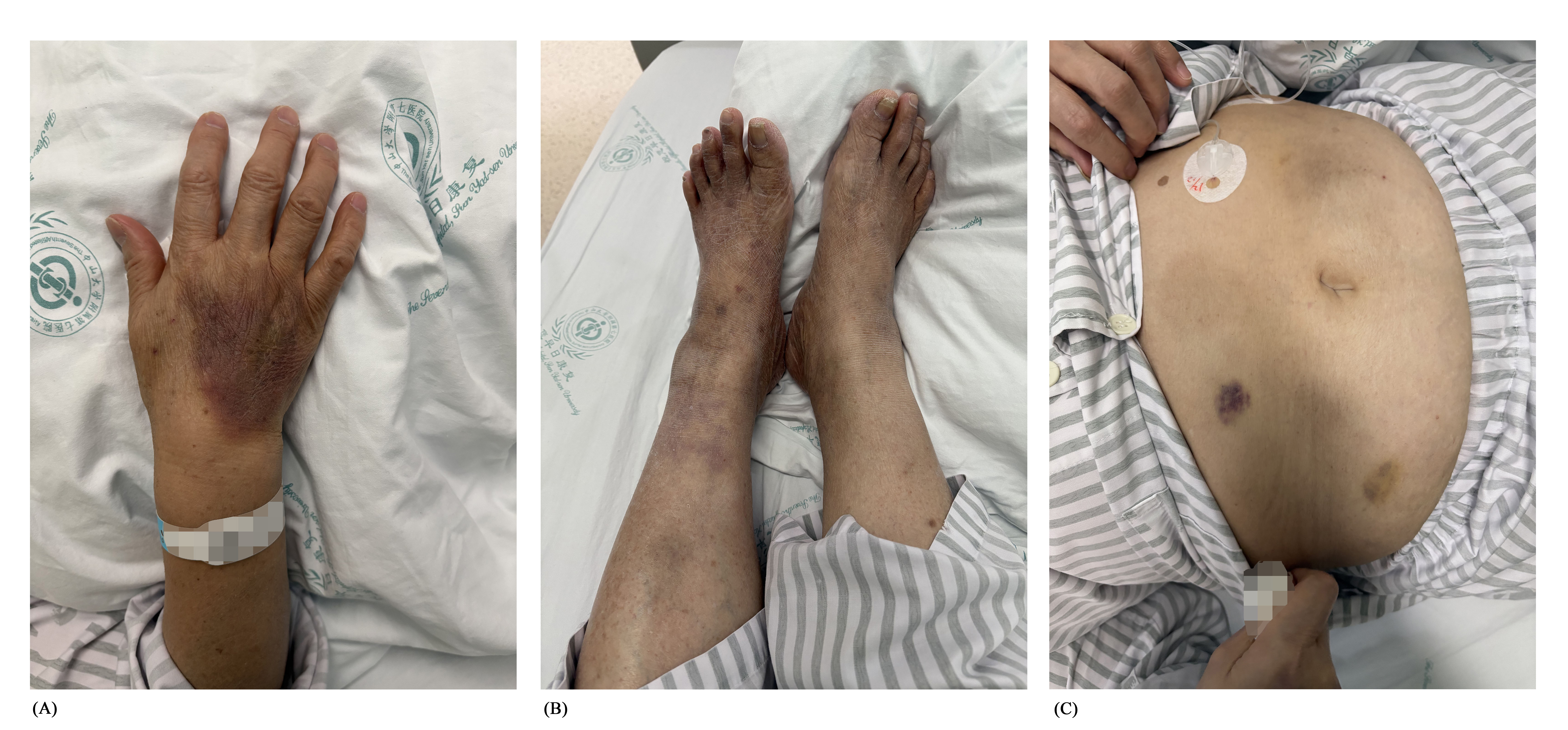

Supplement: Supplementary file 2 [file Image1.jpeg]
