# Supplementary material for: Case report: A case of ITP-like thrombocytopenia induced by denosumab
Source: Front Pharmacol. 2026 Apr 29;17:1723393. doi: 10.3389/fphar.2026.1723393 (PMC13167528; doi:10.3389/fphar.2026.1723393)
Supplement: Supplementary file 3 [file Table2.docx]

| **Causality Term** | **Assessment Criteria** |
| --- | --- |
| **Certain** | • Event or laboratory test abnormality, with **plausible time relationship** to drug intake • **Cannot be explained** by disease or other drugs • Response to withdrawal **plausible** (pharmacologically, pathologically) • Event definitive pharmacologically or phenomenologically (i.e., an objective and specific medical disorder or a recognized pharmacological phenomenon) • Rechallenge satisfactory, if necessary |
| **Probable / Likely** | • Event or laboratory test abnormality, with **reasonable time relationship** to drug intake • **Unlikely** to be attributed to disease or other drugs • Response to withdrawal **clinically reasonable** • Rechallenge **not required** |
| **Possible** | • Event or laboratory test abnormality, with **reasonable time relationship** to drug intake • **Could also be explained** by disease or other drugs • Information on drug withdrawal may be **lacking or unclear** |
| **Unlikely** | • Event or laboratory test abnormality, with a time to drug intake that makes a relationship **improbable** (but not impossible) • Disease or other drugs provide **plausible explanations** |
| **Conditional / Unclassified** | • Event or laboratory test abnormality • **More data needed** for proper assessment, or • Additional data under examination |
| **Unassessable / Unclassifiable** | • Report suggesting an adverse reaction • **Cannot be judged** because information is insufficient or contradictory • Data cannot be supplemented or verified |

Note: The usual approach is to choose one of the causality terms and test whether the various criteria fit the content of the case report. All assessment criteria should be reasonably complied with to assume a category. The system is designed for the assessment of single case reports of adverse drug reactions or drug-drug interactions.
